# The Arabidopsis Rho of Plants GTPase ROP1 Is a Potential Calcium-Dependent Protein Kinase (CDPK) Substrate

**DOI:** 10.3390/plants10102053

**Published:** 2021-09-29

**Authors:** Dalma Ménesi, Éva Klement, Györgyi Ferenc, Attila Fehér

**Affiliations:** 1 Institute of Plant Biology, Biological Research Centre of the Eötvös Lóránd Research Network, 6726 Szeged, Hungary; menesi.dalma@brc.hu (D.M.); ferenc.gyorgyi@brc.hu (G.F.); 2 Laboratory of Proteomics Research, Biological Research Centre of the Eötvös Lóránd Research Network, 6726 Szeged, Hungary; klement.eva@brc.hu or; 3 Single Cell Omics ACF, Hungarian Centre of Excellence for Molecular Medicine, 6726 Szeged, Hungary; 4 Department of Plant Biology, University of Szeged, 6726 Szeged, Hungary

**Keywords:** *Arabidopsis thaliana*, AGC kinase, calcium-dependent protein kinase, CDPK, GTP-binding protein, post-translational modifica-tion, phosphorylation, ROP GTPase

## Abstract

Plant Rho-type GTPases (ROPs) are versatile molecular switches involved in a number of signal transduction pathways. Although it is well known that they are indirectly linked to protein kinases, our knowledge about their direct functional interaction with upstream or downstream protein kinases is scarce. It is reasonable to suppose that similarly to their animal counterparts, ROPs might also be regulated by phosphorylation. There is only, however, very limited experimental evidence to support this view. Here, we present the analysis of two potential phosphorylation sites of AtROP1 and two types of potential ROP-kinases. The S74 site of AtROP1 has been previously shown to potentially regulate AtROP1 activation dependent on its phosphorylation state. However, the kinase phosphorylating this evolutionarily conserved site could not be identified: we show here that despite of the appropriate phosphorylation site consensus sequences around S74 neither the selected AGC nor CPK kinases phosphorylate S74 of AtROP1 in vitro. However, we identified several phosphorylation sites other than S74 for the CPK17 and 34 kinases in AtROP1. One of these sites, S97, was tested for biological relevance. Although the mutation of S97 to alanine (which cannot be phosphorylated) or glutamic acid (which mimics phosphorylation) somewhat altered the protein interaction strength of AtROP1 in yeast cells, the mutant proteins did not modify pollen tube growth in an in vivo test.

## 1. Introduction

Rho family GTPases are implicated in a wide range of basic cellular processes including cell morphology, polarity, motility, division, and defence [1]. These evolutionarily conserved molecules belong to the superfamily of Ras-type small GTP-binding proteins serving as two-state molecular switches [2]. Having intrinsic capabilities for GTP-hydrolysis as well as GDP-to-GTP exchange, they cycle between GTP-bound and GDP-bound conformations [3]. It is the GTP-bound “active” state in which they become attached to the plasma membrane and interact with downstream effector molecules to control and coordinate various cellular events [4]. Hydrolysis of the bound GTP renders the molecule inactive leaving the membrane and releasing the effector(s). The cycle is closed and restarts when the bound GDP is exchanged for GTP. The intrinsic GTP hydrolysis and GDP-to-GTP exchange rates of Rho proteins are very low [5]. Three classes of regulatory proteins facilitate and govern the GTPase cycle [5,6]: the guanine nucleotide exchange factors (GEFs) promote GDP dissociation and thus the re-binding of GTP; the GTPase accelerator proteins (GAPs) enhance GTP hydrolysis; the guanine nucleotide dissociation inhibitors (GDIs) prevent GDP-to-GTP exchange and sequester the GTPase in the cytoplasm. These regulators mediate and integrate various upstream signals controlling the GTPase cycle and thus the downstream signalling events [6,7,8,9].

Although Rho-type GTPases are evolutionarily conserved in eukaryotes [7], they can be classified into subfamilies differentially represented in fungi, animals, or plants [8]. While yeast Rho-type G-proteins belong to either the Cdc42 or Rho subfamilies, in mammals, they form the three so-called “classic” Rho-type GTPase families such as Cdc42-like, Rho-like, and Rac-like, and some later-discovered relatives, the RhoBTB and Rnd subfamilies [9]. Plants have only a single and unique subfamily, designated as Rho-of-plants or ROP GTPases (sometimes also referred as plant Rac GTPases based on their closest similarity to animal Rac proteins) [10].

**Figure 1:**
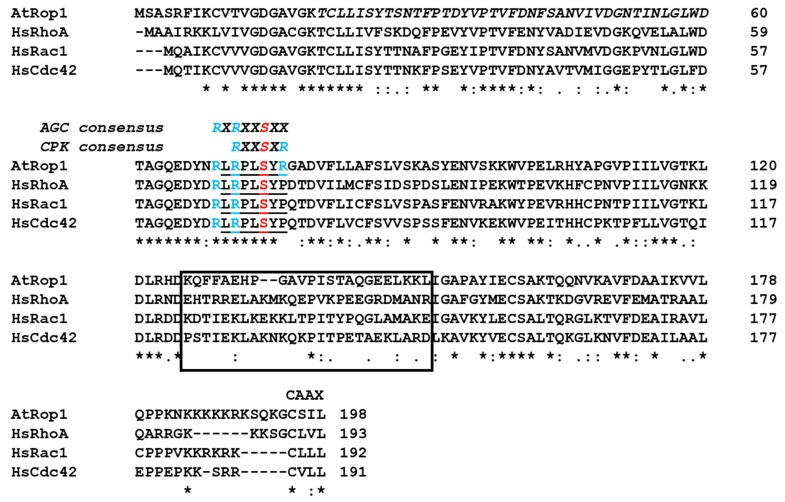
Sequence comparison of the ROP1 GTPase of *Arabidopsis thalina* (AtROP1) with the *Homo sapiens* Rho-type GTPases HsRhoA, HsRac1, and HsCdc42. The evolutionarily conserved AGC kinase phosphorylation site overlapping with the phosphorylation consensus site of plant-specific calcium-dependent protein kinases (CPKs) is indicated above the underlined corresponding sequence of the G-proteins. Conserved Arg residues surrounding the phosphorylation site S74 of AtROP1 are highlighted in blue. The less-conserved Rho-insert region is boxed. The conserved C-terminal CAAX motif is also indicated. * indicates evolutionary conserved residues.

Despite the evolutionarily conservation of all Rho-type GTPases, plant ROPs and their partners have many unique characteristics [10,11,12,13]. All Rho-type G-proteins have a specific region, the so-called “Rho insert region”, a helical structure followed by a short loop extending out from the surface allowing interactions with regulators or effectors. This region is strikingly different in ROPs in comparison to other Rho-like subfamilies, supporting the view that they have unique partners [11]. Indeed, although many Cdc42/Rac/Rho GTPase effectors are missing from plants, ROPs acquired their own specific downstream partners during evolution [10,12,13,14]. As their regulators are concerned, RopGAPs have a unique domain combination strengthening their specific interaction with ROPs [11,15], while the RopGEFs are entirely plant-specific proteins carrying the “plant-specific ROP nucleotide exchanger” (PRONE) domain not present outside of the plant kingdom [11,16]. In agreement, the PRONE domain was shown to be inefficient in the promotion of the GDP-to-GTP exchange on the human Rac1 GTPase; it accepts only ROPs as substrates [16,17]. The specificity of the PRONE domain towards ROPs is mainly due to two plant-specific amino acid residues, asparagine 68 (N68) and arginine 76 (R76) (based on Arabidopsis ROP4 numbering; Figure 1), close to the “switch II” region [17], involved in the GTP-binding-dependent conformation switch of G-proteins [18]. These two residues are conserved in all plant ROPs but do not occur in any other Rho-type GTPases [11,17]. It was shown that mutating the P71 residue (corresponding to R76 in ROP4) of human Rac1 to arginine was sufficient to turn the human Rac1 protein into a PRONE-accepted substrate [17].

Rho GTPases, like almost all Ras-related proteins, contain a C-terminal cysteine-containing motif (e.g., CAAX, where the two A residues are aliphatic amino acids and X represents any amino acid; Figure 1) as the prenylation site required for their membrane attachment and function [6,19]. Plant ROPs can be subdivided into two main groups (Type I and Type II) differing among others in their extreme C-terminal motif allowing posttranslational lipid modifications [11]. In addition to prenylation, Rho GTPases are subjected to various other post-translational modifications including phosphorylation, ubiquitination, acylation, etc., modifying their interactions with regulators or effectors, their stability, and localisation [6]. Thus, the precise spatiotemporal activation of Rho GTPases depends on many factors including direct or indirect interactions with protein partners.

Phosphorylation of human Rho-type GTPases by various protein kinases has been widely reported [6]. Phosphorylations close to sites of lipid modifications and membrane targeting were reported to modulate the intracellular location of RhoU and Rac1 proteins [20,21]. Phosphorylations within the GTPase domain were found to interfere with the GDP–GTP cycle and the signalling activity of the Rac1 GTPase [22,23]. Phosphorylations of different surface residues were shown to directly influence RhoA’s interaction with effectors [24] and regulators [25], respectively. Phosphorylation controls the ubiquitination and degradation of the Rac1 GTPase [26]. The accumulating evidence supports the view that phosphorylation of Rho-type GTPases has an important regulatory role, fine tuning their signalling activity [6].

Interestingly, the ROP-specific R76 residue that is required for the PRONE-mediated nucleotide exchange is close to an evolutionarily conserved serine residue (S71 in human Rac1 or S74 in Arabidopsis Rop4, respectively; Figure 1). This serine residue of human Rac1 was shown to be phosphorylated by the Akt kinase, affecting its nucleotide-binding and correspondingly its signalling function [23]. The same phosphorylation strengthened the interaction of Rac1 with 14-3-3 proteins [27]. Furthermore, phosphorylation of Rac1 S71 by AKT was shown to facilitate its ubiquitination and subsequent proteasomal degradation of Rac1 [26]. The Akt recognition sequence RXRXXS71/74XX is evolutionarily conserved in all plant ROPs (Figure 1). Therefore, the potential role of S74 phosphorylation in the regulation of ROP GTPase signalling was hypothesized [17,28]. In order to investigate this possibility, the S74 residue of the *Medicago sativa* ROP6 (MsROP6) GTPase was mutated to alanine (S74A) or glutamic acid (S74E) [28]. The latter mutation, due to the negative charge of the E residue, mimicked the phosphorylated state of S74. It was found that unlike in the case of Rac1, the nucleotide-binding ability of MsROP6 was not affected by these mutations. However, the phosphorylation-mimic S74E mutation specifically interfered with the PRONE-mediated activation of the GTPase as well as by the activation of a downstream effector kinase [28]. These experimental data strengthened the view that ROPs might also be subjected to direct regulation by phosphorylation [12]. Recently, the in vitro phosphorylation of the barley RACB GTPase by its own downstream effector kinase HvRBK1 was reported at several positions excluding S74; however, the functional significance of the phosphorylation of these sites remained obscure [29].

Here, we report that although our approaches to identify Akt-related plant AGC kinases potentially phosphorylating the S74 residue of plant ROPs failed, we could demonstrate that plant calcium-dependent protein kinases (CPKs) accept the Arabidopsis ROP1 GTPase as in vitro substrate in a conformation-dependent way. The phosphorylation takes place at several Ser/Thr residues, excluding S74, and the phospho-mimetic mutation of one of these residues affects the protein–protein interaction capability and the function of the GTPase. These data open new avenues to investigate the interlink between kinase and ROP GTPase signalling pathways in plants.

## 2. Results

### 2.1. The Arabidopsis ROP1 GTPase Does Not Serve as In Vitro Substrate of the AGC 1.7 Kinase

**Figure 2:**
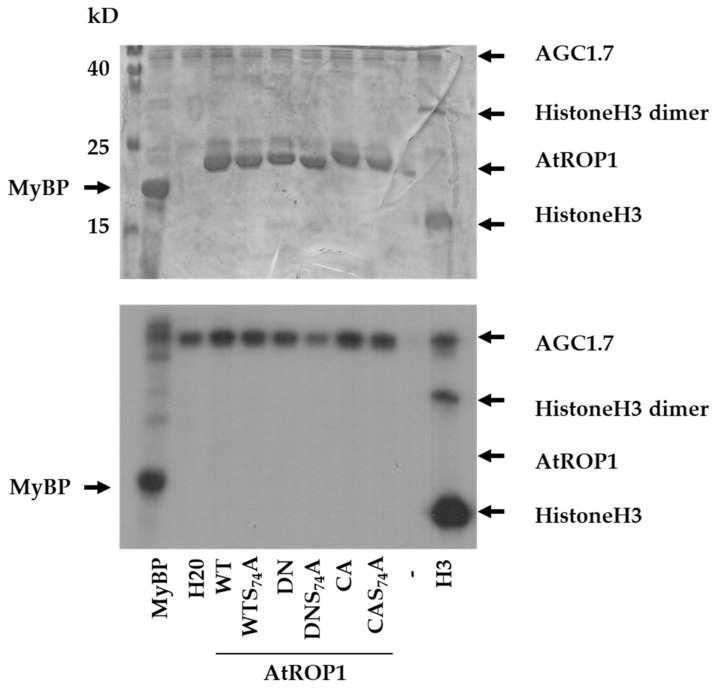
In vitro protein phosphorylation assay using the Arabidopsis AGC1.7 kinase and various substrates: the pig myelin basic protein (MyBP), human histone H3 (H3), and various forms of the AtROP1 GTPase (WT—wild type; DN—dominant negative; CA—constitutive active; S74A—mutation of serine74 to alanine). The Coomassie Brilliant Blue (CBB)-stained gel is shown on the upper part, and its autoradiograph (P32) is below. The approximate sites of the proteins on the gel/autoradiogram are shown by arrows. Molecular weights are indicated in kD beside the molecular weight marker in the stained gel.

We have previously demonstrated that the S74E phosphorylation-mimic mutation of the *Medicago sativa* ROP6 protein interfered with the regulation and consequently with the in vitro as well as *in planta* function of the GTPase [28]. The S74 residue is within an evolutionarily conserved phosphorylation site motif of AGC-type protein kinases (Figure 1). Moreover, the human Akt kinase belonging to this class of protein kinases [30] is known to regulate the human Rac1 GTPase phosphorylating the protein at this site [23,26]. To investigate the possibility that plant AGC-type protein kinases also phosphorylate and regulate the Rac1-like plant ROP GTPases, we produced and purified Arabidopsis AGC1.7 kinase (AT1G79250; Q1PFB9) and the Arabidopsis ROP1 GTPase (At3g51300; P92978) proteins. The AGC1.7 kinase was selected based on its proven role in the polar growth of pollen tubes controlled by the ROP1 GTPase [31,32]. To ensure and strengthen its in vitro kinase activity, a phosphorylation mimic mutation (S379E) was introduced to the T-loop of AGC1.7 mimicking the upregulation of kinase activity by 3-phosphoinositide-dependent kinase 1 (PDK1) phosphorylation [33,34,35,36]. Since the conformation of ROP1 is dependent on nucleotide-binding that may affect its phosphorylation, dominant negative (DN with the T20N mutation locking the protein in the GDP-bound state) and constitutive active (CA with the G15V mutation resulting in the GTP-bound conformation) proteins were also used as substrates apart from the wild-type one (WT). Moreover, mutant versions where the S74 residue was mutated to alanine (S74A) were also included. The in vitro kinase assay showed that the AGC 1.7 kinase was active (could phosphorylate the myelin basic protein as well as histone H3 substrates) but was unable to phosphorylate the ROP1 protein at any site, irrespectively of the GTPase’s conformation (Figure 2).

In order to test whether animal AGC kinases can phosphorylate the evolutionarily conserved phosphorylation site, the commercially available murine cAMP-dependent protein kinase (PKA) catalytic subunit was used in a similar kinase assay. It was found that although this kinase domain was able to phosphorylate the AtROP1 protein, this phosphorylation was neither dependent on the conformation nor the presence of the S74 residue of the GTPase (Figure S1). Therefore, it is unlikely that the potential phosphorylation of Rop1 S74 is mediated by an AGC-type kinase.

### 2.2. The Arabidopsis Calcium-Dependent Protein Kinases CPK17 and CPK34 Can In Vitro Phosphorylate the ROP1 GTPase Dependent on Its Conformation but Not at the S74 Residue

The amino acid residues surrounding S74 of AtROP1 can also serve as a recognition site for the plant-specific calcium-dependent protein kinases (CPKs) [37] (Figure 1). Moreover, members of the Arabidopsis CPK family, CPK17 and CPK34, were shown to be required for the polarized growth of pollen tubes [38].

**Figure 3:**
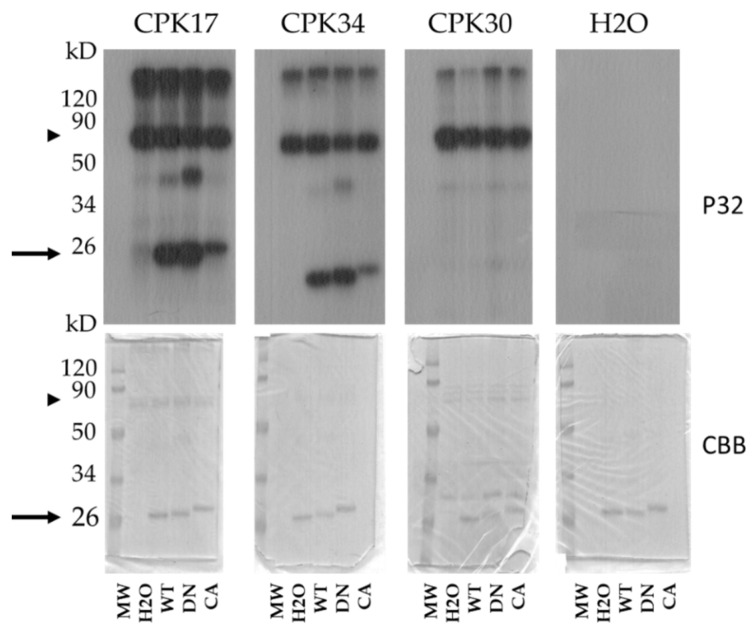
In vitro protein phosphorylation assays using the Arabidopsis CPK17, CPK 34, and CPK30 protein kinases and GDP-GTP-bound mutant forms of the AtROP1 GTPase (WT—wild type; DN—dominant negative; CA—constitutive active). The Coomassie Brilliant Blue (CBB)-stained gels are shown on the lower part, and their autoradiographs (P32) are above. The approximate position of the AtROP1 proteins on the gel/autoradiogram are shown by arrows and that of the CPK kinases by arrowheads. kD—molecular weight in kiloDaltons.

**Figure 4:**
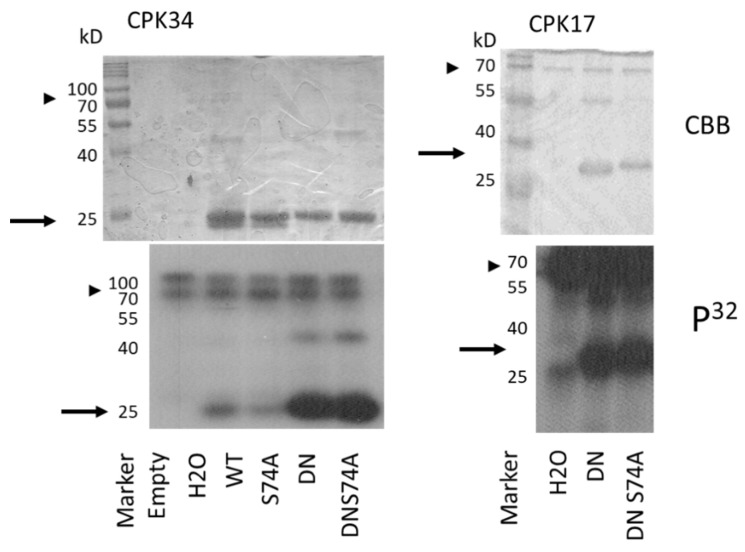
In vitro protein phosphorylation assays using the Arabidopsis CPK17 CPK 34 kinases and wild-type or mutant forms of the AtROP1 GTPase (WT—wild type; DN—dominant negative; S74A—serine-to-alanine at position 74). The Coomassie Brilliant Blue (CBB)-stained gels and their autoradiographs (P32) are shown. The approximate position of the AtROP1 proteins on the gel/autoradiogram are shown by arrows, and that of the CPK kinases by arrowheads. kD—molecular weight in kiloDaltons. H2O stands for no substrate reaction.

In the Arabidopsis pollen tube, there is a feedback regulation between the AtROP1 GTPase and a tip-localised calcium gradient [39]. Therefore, it was speculated that CPK-dependent phosphorylation of AtROP1 might contribute to this feedback mechanism. In order to test this possibility, CPK17, CPK30, and CPK34 protein kinases were produced and purified and used in in vitro kinase activity assays using various forms (WT, DN, and CA) as substrates. It was found that CPK17 and CPK34 but not CPK30 phosphorylated the AtROP1 protein in a conformation-dependent way: the phosphorylation of the DN version was the strongest, followed by the WT and the weakly phosphorylated CA GTPase (Figure 3). To determine whether the phosphorylation takes place on the S74 residue, the S74A mutant versions of the WT and/or DN GTPase were also tested as substrates. As shown on Figure 4, the CPK17 and CPK34 kinases could phosphorylate the S74A mutant versions of the GTPase, indicating that it phosphorylates the AtROP1 at other site(s), not S74.

### 2.3. CPKs Phosphorylate the S97 Residue of AtROP1 but the S97E Phosphomimic Mutation Has No Significant Effect on the Function of It

**Figure 5:**
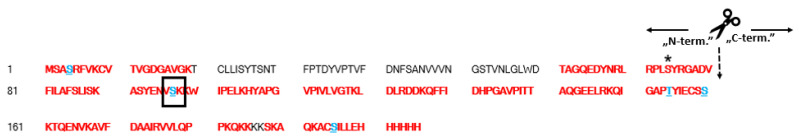
Phosphorylation of Arabidopsis ROP1 by CPK17. CPK17-dependent in vitro phosphorylated sites of AtROP1 as determined by mass spectrometry (MS). The detected sequence is shown in red. Phosphorylated residues in the identified peptides are in blue. The non-phosphorylated S74 residue is indicated by *. The S97 residue represented by most of the phosphopeptides (Table S1) is boxed. The scissors symbol indicates where the sequence was dissected to separately express N- and C-terminal regions, respectively.

**Figure 6:**
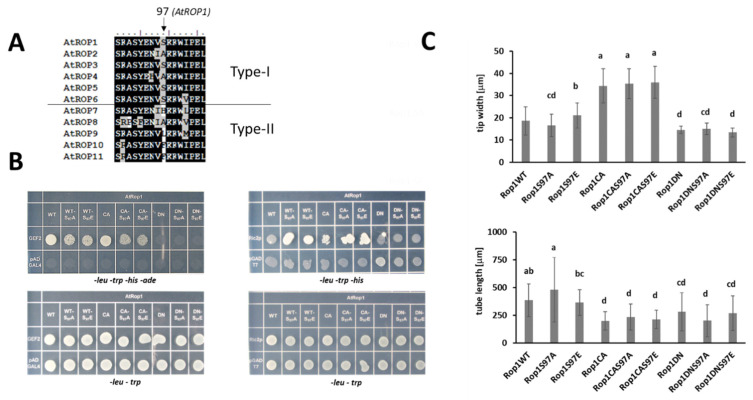
Effect of the S97A or S97E mutations on ROP1 function. (**A**) The S97 residue is conserved only in four Type-I ROPs. (**B**) Both mutations similarly affect the interaction of ROP1with GEF2 or RIC2 in the yeast two-hybrid system (leu- trp-selection for positive transformation; his- ade- selection for interaction). (**C**) Effect of the transient expression of wild-type and mutant YFP-fused ROP1 versions driven by the Lat52 pollen-specific promoter on the growth of tobacco pollen tubes. Tip width and tube length were measured for 20–20 fluorescent pollen tubes. Averages and standard deviations are shown. Different letters mean statistically significant difference (*p <* 0.05; one-way analysis of variance (ANOVA) following Duncan’s multiple range tests). WT—wild type; CA—constitutive active; DN—dominant negative.

To determine the site of phosphorylation, kinase assays were made using the CPK34 kinase in the presence of “cold” ATP and the WT and DN forms of the AtROP1 protein as substrate. The phosphorylated proteins were cut out of CBB-stained gels and subjected to trypsin digestion followed by partial acidic cleavage, phosphopeptide enrichment, and mass spectrometry analysis to detect phosphopeptides. Several serine and one threonine residues were found to be phosphorylated in both ROP1 versions (Figure 5, Table S1). The S97 serine was abundantly detected as phosphorylated. This residue is, however, conserved only in those Type-I ROP proteins (ROP1, ROP3, ROP5, and ROP6; Figure 6A) that are highly expressed in pollen [40,41].

Nevertheless, we mutated it into alanine (S97A) or glutamic acid (S97E) to test whether its phosphorylation might have an effect on ROP1 function. Beside introducing into the wild type, the S97 phosphorylation-related mutations were combined with the constitutive active (CA) and dominant negative (DN) mutations. The interactions of the mutant versions with an upstream regulator GEF2 and a downstream effector RIC2 were tested in the yeast two hybrid system. The S97A and S97E mutations weakened the interaction of WT or CA ROP1 with GEF2 but strengthened it with RIC2. Since both the S97A and the phospho-mimic S97E mutation affected the interactions in a similar way (Figure 6B), the results indicated the significance of S97 for protein interactions of ROP1 but were not conclusive considering the role of its potential phosphorylation. To have further evidence, the ROP1 protein and its mutant versions were transiently expressed in pollen tubes, and their effect on the growth and polarity (tip width) of the tube were measured. Active ROP1 was expected to increase tip width while the protein locked in inactive conformation to shorten tube length. The WT, CA, and DN versions of ROP1 influenced pollen tube growth as expected, irrespective of S97 mutations (Figure 6C). This indicated little or no effect of the potential phosphorylation of S97 on *in planta* ROP function.

### 2.4. The CPK17/34 Kinases Can Phosphorylate the ROP1 Protein at Several Sites In Vitro

**Figure 7:**
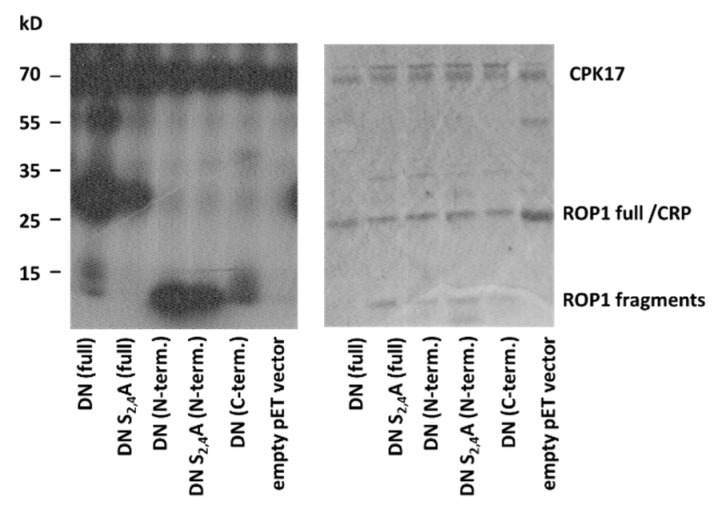
In vitro kinase assay using the CPK17 kinase and the DN mutant ROP1 GTPase either in full length, or its N- or C-terminal regions, respectively. The S2 and S4 residues were mutated (S2G S4A) to investigate the potential phosphorylation of the region that could not be detected by MS. Since an *E. coli* cAMP-activated global transcriptional regulator CRP (NCBI# 15803871, MW 23.8 kDa) co-purified with the 6xHIS ROP1 protein with about the same size, an empty vector control was included into the experiment.

The other sites phosphorylated by CPKs on ROP1 resided in the N-(S4) or C-terminal regions (T154, S160, S195) of the protein. It has to be mentioned that the region between T20 and D60 was not detected by MS, probably because this region contains neither tryptic cleavage site nor basic amino acids promoting ionisation (Figure 5). To decide whether the studied CPKs have preference for the N- or C-terminal region of ROP1, we expressed the 6xHIS-tagged first and second halves of the protein separately in bacteria and used the purified fragments in in vitro kinase reactions with CPK17. Furthermore, we included the S2A S4A mutations into the N-terminal region to be able to decide whether the T20-to-D60 region might be phosphorylated by CPKs. The results of the in vitro assays indicated that it is the N- rather than the C-terminal region of the AtROP1 protein that is efficiently phosphorylated by CPK17 but not only at the S4 site (Figure 7). Therefore, it was clear that the T20-D60 region should contain at least one additional CPK17 phosphorylation site.

## 3. Discussion

Rho-type GTPases of animals are well-known to be integrated into both upstream and downstream kinase-related signal transduction pathways [42]. They are directly regulated by [6], and they directly regulate several protein kinases [43]. However, much less is known about the link of the related plant-specific ROP G-proteins to kinases since none of the Rho-interacting kinase families that were described in animals exist in plants [12]. As kinases downstream of ROPs are considered, the members of the Arabidopsis Receptor-like Cytoplasmic Kinase Class VI GroupA (RLCK VI_A) and related proteins from other species are good candidates as direct ROP signalling partners [44,45,46,47]. As the upstream regulation of ROPs by kinases is considered, it is well-accepted that several plant receptor-like kinases (RLKs) control ROP GTPase signalling through RopGEFs, the activators of ROPs [12,48]. Evidence of plant kinases that phosphorylate ROPs and thus directly influence their functions is still missing. We previously described that one of the phosphorylation sites of animal Rho-type small GTPases is conserved in plant ROPs as well and that phosphomimic mutation of the corresponding serine residue (S74 in AtROP1; see Figure 1) specifically affected the RopGEF-mediated activation of Medicago/Arabidopsis ROP GTPases both in vitro and in vivo [28]. This site is phosphorylated in the animal Rac1 GTPase by the Akt protein kinase [23], the plant relatives of which belong to the large family of plant AGC kinases [33]. Two members of this kinase family (AGC 1.5 and 1.7) were shown to be involved in the regulation of pollen tube polarity [32], a process controlled by ROP GTPases [31]. Therefore, it was reasonable to suppose that ROPs might serve as substrates of these kinases. However, our in vitro kinase assays using one of these Arabidopsis AGC kinases, AGC1.7, indicated no such direct relationship (Figure 2). Recently, it was demonstrated that AGC1.5 rather controls ROP-mediated pollen tube polarity via the phosphorylation of RopGEFs [49].

The AGC-type kinase phosphorylation consensus sequence around S74 of AtROP1 (RXRXXS) due to a plant-specific conserved arginine residue overlaps with one of the predicted phosphorylation site of calcium-dependent protein kinases (CPKs) (RXXSXR; [37]. It is well demonstrated that the tip-localised oscillating calcium gradient is central to maintaining pollen tube polarity and growth in concerted action with ROP GTPases [31,39,50]. Several members of the large CPK family have been implicated in the control of pollen tube growth [51,52]. Among others, it was shown that CPK17 and 34 are mutually required to maintain the proper rate of pollen tube growth, and they are also involved in its guidance towards the ovules [38]. Based on the above, we tested whether CPKs, namely CPK17 and/or CPK34, can phosphorylate ROP1. We could establish that both of these kinases but not CPK30, not being implicated in pollen tube growth control [53], could efficiently phosphorylate ROP1 in vitro (Figure 3). Moreover, the GTPase was less amenable for phosphorylation if it was locked in the GTP-bound constitutive active conformation by the G15V mutation in comparison either to the wild type protein or its dominant negative (T20N) GDP-bound mutant form (Figure 3). However, the phosphorylation was not influenced by the S74A mutation, indicating that the investigated CPKs phosphorylate AtROP1 at other sites.

To determine the potential phosphorylation sites, in vitro CPK17-phosphorylated AtROP1 was subjected to phosphopeptide analysis by mass spectrometry. The analysis indicated that several sites of ROP1 are phosphorylated by CPK17 in vitro (Figure 5). Among these sites, phosphorylation of S97 was abundantly detected. Mutating this site to either the phospho-mimic glutamic acid (S97E) or to the non-phosphorylatable alanine (S97A) affected in a similar manner the binding of AtROP1 to proteins representing upstream regulators and downstream effectors, respectively (Figure 6B). This indicated that the role of the S97 residue in protein–protein interactions is independent from its phosphorylation state. It is supported by the fact that this residue is not conserved in all Arabidopsis ROPs (Figure 6A), e.g., in AtROP2 and AtROP6 there is an alanine at the 97th position. The presence of alanine or serine residue at this position of Type I ROPs might influence their binding strength/specificity to signalling partners. The residues at the 97th position of Type I ROPs are conserved across plant species, supporting this view. This residue was shown also to be in vitro phosphorylated in a barley ROP GTPase, HvRACB, by ROP Binding Kinase 1 (RBK1) [29]. This kinase belongs to receptor-like cytoplasmic kinases that serve as ROP GTPase effectors [12,45], raising the possibility for feed-back regulation of ROP signalling via RBK1. The in vitro phosphorylation of HIS:AtROP4 and HIS:AtROP6 by AtRBK1 (also named as AtRLCK VI_A4; [54]) was also reported [55]. However, since RLCK VI_A kinases were found to efficiently phosphorylate the linker of the HIS-tag produced by pET vectors during protein expression [44], these data are not conclusive.

All attempts to identify the in vivo phosphorylation of HvRACB at S97 have failed [29]. In agreement, our functional assay expressing the S97A or S97E mutant AtROP1 in tobacco pollen tubes (Figure 6C) could not verify the in vivo significance of a potential S97 phosphorylation. One may suppose that since S97 is surface exposed [29], it might be accessible for phosphorylation by several kinases in vitro while protein–protein interactions might mask the site in vivo. It is worth mentioning that the phospho-sites mapped on the AtROP1 and HvRACB GTPases after in vitro phosphorylation by HvRBK1 and AtCPK17, respectively, only partly overlap (at S97 and S160; (Figure S2), indicating kinase specificity of ROP phosphorylation even in vitro. Although kinases have preference for certain amino acid sequences (motifs) surrounding the phosphorylation site, these preferences are often rather weak. For example, for plant CPKs, seven different phosphorylation site consensus motifs have been reported, including some very simple ones [56]. Plant CPKs classified into 12 subgroups have differential affinities for the various motifs underlying their specificities [53,57]. In agreement, only the pollen-expressed CPK17 and CPK34 but not CPK30 could efficiently phosphorylate AtROP1 in vitro.

Dissecting the AtROP1 protein into two halves, using them as substrates in two parallel kinase assays, indicated that both parts are phosphorylated by CPK17 but the N-terminal part (AtROP1 1-80) exhibited a stronger phosphorylation signal than the C-terminal part including most of the sites identified by MS (S97, T154, S160, S195; Figure 7). When the S4 residue implicated in CPK17 phosphorylation by MS was mutated to alanine, the signal hardly diminished indicating further potential phosphorylation sites at the N-terminal part of AtROP1 (Figure 7). Since the region of AtROP1 (and other ROPs including HvRACB) between T20 and D60 lacking suitable cleavage sites and ionisable amino acids is not amenable for MS analysis, we suppose that it contains one or more CPK17 target site(s).

Although from the experiments described in this paper and in the work of Weiss et al. [29], it is clear that ROPs can be specifically phosphorylated by various kinases in vitro; the evidence for in vivo ROP phosphorylation is scarce. Recently, S138 of the Type II ROPs, AtROP10 and AtROP11, were shown to be phosphorylated *in planta* in an untargeted phosphoproteomic approach [58]. AtROP1 was found to be phosphorylated in a comprehensive analysis of circadian-regulated protein phosphorylation events in Arabidopsis seedlings [59] at its N-terminal region at S2, S4, and T11 residues. The kinase(s) phosphorylating these sites in vitro are not known. Based on our results, CPK17 might phosphorylate S4, but neither the S2 nor T11 residues were found to be phosphorylated by this kinase in vitro. These types of approaches are very useful but provide little information about the biological relevance of the phosphorylation event. Moreover, as is the case with plant ROPs, certain regions of proteins might not be amenable for mass spectrometric analysis or might be underrepresented or context-dependent, limiting the comprehensiveness of such databases. To link phosphoproteomic patterns to given kinases, the phosphoproteomic studies with wild-type plants/tissues should be repeated in various kinase mutant backgrounds; however, such attempts will detect not only direct but indirect phosphorylation changes as well.

Calcium and CPKs are intimately linked to ROP GTPases [12,31,38,39,50,51,60,61]. Experimental evidence indicates that calcium signalling converges on ROP GTPases via the phosphorylation of various ROP-regulators such as RopGAPs [62], RopGEFs [63], or RopGDIs [64]. Our in vitro studies suggest that certain CPKs including CPK17 and CPK34 might directly phosphorylate ROPs themselves at several potential sites. One of these sites in AtROP1 (S97) has been tested, but the studies indicated no significant biological relevance of the in vitro results. Only individual testing and functional analysis of further potential sites, including those that could not be revealed by MS analysis, could answer the question whether the direct link between CPKs and ROPs does indeed exist.

## 4. Materials and Methods

### 4.1. Molecular Cloning

Cloning of AtROP1 and its DN (T20N) and CA (G15V) mutant versions into the bacterial protein expression vector pET26b has been described earlier (Dorjgotov et al., 2009; note: the periplasm-targeting sequence was removed, and the 6xHIS-tag is C-terminal with a short linker that has no any potential phospho-sites). The S97A and S97E mutations were introduced by the overlap extension polymerase chain reaction approach (Urban et al., 1997) using the 5′ AtR1 NdeI forward and the AtROP1S97A BamHI Rev or AtROP1S97E BamHI Rev primers (Table S2), respectively, on the wild-type, DN, and CA pET26b AtROP1 constructs as templates. The NdeI/BamHI fragments were than exchanged by standard molecular cloning procedures. The full length wild-type and various mutant sequences were PCR amplified by the 5′ AtR1 NdeI forward and AtRop1-SalI(lm) Rev reverse pimers to insert at the NdeI and SalI sites of the pGADT7-Dest vector, and by the AtRop1 Fw (SalI + 2ncl) and AtROP1 Rev (Kpnl) primers to clone into the pWEN240 YFP [65] pollen expression vector (for primer sequences see Table S2). The CPK 17 and 34 cDNA sequences were amplified by PCR using appropriate 5′ and 3′ primers (Table S2) and cloned into NdeI/NotI sites of the pET28a vector for bacterial protein purification. The CPK30 cDNA was moved from GST-CPK30cDNA-6xHis into pET28a as a NcoI-SacI insert. GEF2 and RIC2 cDNAs were amplified using appropriate 5′ and 3′ primers (Table S2) and cloned into the EcoRI and XhoI sites of the yeast two-hybrid vector pGBKT7. Constructs carrying the full-length cDNA clones serving as PCR/cloning templates were obtained from the Arabidopsis Biological Resource Center (ABRC; http://www.Arabidopsis.org/ (last accessed on 15 September 2021).

For the polymerase chain reaction (PCR), Phusion high-fidelity polymerase was used (Thermo Fisher Scientific, Waltham, MA, USA), according to the manufacturer’s instructions. The constructs were verified by Sanger sequencing of the inserts.

### 4.2. Protein Purification, In Vitro Kinase Assay

Plasmid constructs for protein expression were transformed into BL21(DE3)Rosetta (Novagen part of Merck KGaA, Darmstadt, Germany) or ArcticExpress(DE3)RIL (Agilent technologies, Santa Clara, CA, USA) competent cells, respectively. Proteins were induced to be expressed using 1mM IPTG (Thermo Fisher Scientific, Waltham, MA, USA) during an incubation for 45 min to 12 h according to the competent cell’s description. The expressed proteins were purified with HIS-Select Nickel Affinity Gel (Sigma-Aldrich, St.Louis, MO, USA) or Ni-IDA Agarose gel (Biontex, Munich, Germany), following the manufacturer’s instructions. Purified proteins were concentrated using Amicon Ultra 0.5 mL filters (Merck Millipore Ltd., Tullagreen Carrigtwohill, T45 KD29 Ireland) and then stored in 10% (*v/v*) glycerol at −20 °C for later use.

Kinase assays were carried out in 20 uL reaction volumes at 25 °C for 30 min by incubating app. 50 pmol substrate (6xHIS-tagged ROP versions) with app. 5 pmol kinase protein (6xHIS-tagged AGC1.7 or CPK17, 34, or 30). The buffer to test AGC1.7 kinase activity included 20 mM Tris-HCl, pH 7.5, 5 mM MgCl_2_, 50 mM NaCl, 10 μM ATP, 1mM DTT, and 2 μCi of [γ-32P]ATP. The assays buffer for CPKs contained 25 mM Tris-HCl, pH 7.5, 10 mM MgCl_2_, 10 μM ATP, 0.45 mM EGTA, 0.55 mM CaCl_2_, and 2 μCi of [γ-32P]ATP. The reactions were stopped by 5xSDS loading buffer, boiled for 5 min, and then the proteins were separated on 12% SDS–polyacrylamide gels. The gels were stained by Coomassie Brilliant Blue, dried, scanned, and subjected to autoradiography to detect the radioactive signals.

### 4.3. Mass Spectrometry

Non-radioactive kinase assays were performed parallelly to determine phosphorylation sites by mass spectrometry. The Coomassie Brilliant Blue stained gel bands were cut and in-gel digested by trypsin. Disulphide bridges of the proteins were reduced by dithiothreitol and free sulfhydryls alkylated by iodoacetamide. Digestion with side-chain-protected porcine trypsin (Promega, Madison, WI, USA) was performed for 4 h at 37 °C. The extracted peptide mixtures were split, and half of the digests was submitted to acidic cleavage by formic acid. Phosphopeptide enrichment was performed by immobilized metal affinity chromatography on magnetic Fe-NTA agarose beads that were prepared from Ni-NTA beads (Qiagen, Germantown, MD, USA) by replacement of Ni^2+^ ions to Fe^3+^ ions. Protein digests were loaded onto the Fe-NTA beads in 0.1% trifluoroacetic acid and 80% acetonitrile, the beads washed with loading buffer, then phosphopeptides eluted by 1% ammonium hydroxide and 50 % acetonitrile, and dried. Digests with or without phosphopeptide enrichment were analysed using LC-MS/MS on a Waters Acquity nanoHPLC (Waters, Milford, MA, USA) on-line coupled to a LTQ Orbitrap Elite mass spectrometer (Thermo Fisher Scientific, Waltham, MA, USA) in data-dependent acquisition mode using HCD and ETD fragmentation. Database search was performed by Protein Prospector (v 5.12.3), first against the complete Swissprot 2014.6.10 database (545388 entries) considering only tryptic peptides, and then against the identified proteins also considering cleavage after aspartic acid and glutamic acid. Precursor mass tolerance was 5 ppm and fragment mass tolerance was 20 ppm for HCD fragments and 0.8 Da for ETD fragments. Carbamidomethylation of cysteine residues was set as fixed modification, and oxidation of methionine residues, pyroglutamic acid formation from peptide N-terminal glutamine residues, methionine loss and acetylation of protein N-termini and phosphorylation of serine, threonine, and tyrosine residues as variable modification. Protein and peptide hits were accepted with a minimum score of 22 and 15 and a maximum E value of 0.01 and 0.05 (proteins and peptides, respectively). Phosphopeptide matches were also inspected. The mass spectrometric analysis of phosphorylation sites was described elsewhere in even more detail [66].

### 4.4. Protein-Protein Interaction

Yeast two-hybrid screening was carried out using the Saccharomyces cerevisiae yeast strain, with AH109 as the host. The yeast strain was co-transformed with pDest-GADT7 (AtRop1 and its mutants) and pDest-GBKT7 or pAD-GAL4 (Rop effectors and regulators) constructs based on the Yeast Protocols Handbook (Clontech, part of Takara Bio, Mountain View, CA 94043 USA, https://www.takarabio.com/documents/User%20Manual/PT3024/PT3024-1.pdf—last accessed 20 July 2021). Transformants were grown on appropriate drop-out media in order to follow up the activation of the ade2 and/or the his3 reporter genes (in the presence of 0-, 1-, 3-, or 10 mM 3-aminotriazole (3-AT) where it was required).

### 4.5. Pollen Transformation and Microscopy

Freshly collected or frozen tobacco (*Nicotiana tabacum*) pollen grains were shaken in liquid pollen germinating medium [67], filtered onto wet filter paper, and then bombarded with gold microcarriers coated with 3 µg plasmid DNA using a helium-driven PDS-1000/He particle delivery system (Bio-Rad, Hercules, CA, USA), as described in [67]. Pollen grains were then transferred onto solid pollen germination media and allowed to germinate at 28 °C for 4 h. After the incubation, they were placed onto cover slips for microscopic analysis. Pollen tubes expressing the pLat52: AtRop1 constructs were tracked by spinning disk confocal microscopy (Visitron, Germany) with ×20 (LUCPlan FL 20× (dry, 0.45NA)), and ×40 (dry, 0.6NA) objectives. During imaging, the laser intensity and the camera exposure settings were kept constant. Pollen tube length and pollen tube tip diameter (15 µm from the tip) were measured with the help of the ImageJ software (http://rsbweb.nih.gov/ij/ (last accessed on 15 September 2021.)) in 20-20 randomly chosen pollen tubes expressing the given transgene. The significance of difference between sets of data was determined by one-way analysis of variance (ANOVA) following Duncan’s multiple range tests; a P value of less than 0.05 was considered significant.

### 4.6. Accession Numbers

ROP1 (AT3G51300); CPK17 (AT5G12180); CPK34 (AT5G19360); CPK30 (AT1G74740), GEF2 (AT1G01700); RIC2 (AT1G27380); AGC1.7 (AT1G79250);

## Data Availability

Not applicable.

## Supplementary Materials

The following are available online at https://www.mdpi.com/article/10.3390/plants10102053/s1: Figure S1: In vitro phosphorylation of AtROP1 by the catalytic domain (CD) of murine PKA, Figure S2: Comparison of sites in vitro phosphorylated by the RBK1 kinase in barley HvRACB GTPase (Weiß et al. 2020) and by the CPK17 kinase in Arabidopsis AtROP1 (this study), Table S1: CPK17 phosphorylated AtROP1 peptides detected by mass spectrometry, Table S2: PCR primer oligonucleotid sequences.
