# Supplementary material for: The Arabidopsis Rho of Plants GTPase ROP1 Is a Potential Calcium-Dependent Protein Kinase (CDPK) Substrate
Source: Plants (Basel). 2021 Sep 29;10(10):2053. doi: 10.3390/plants10102053 (PMC8539224; doi:10.3390/plants10102053)
Supplement: Supplementary file 1 [file plants-10-02053-s001.zip › Figure S1.pptx]

## Slide 1
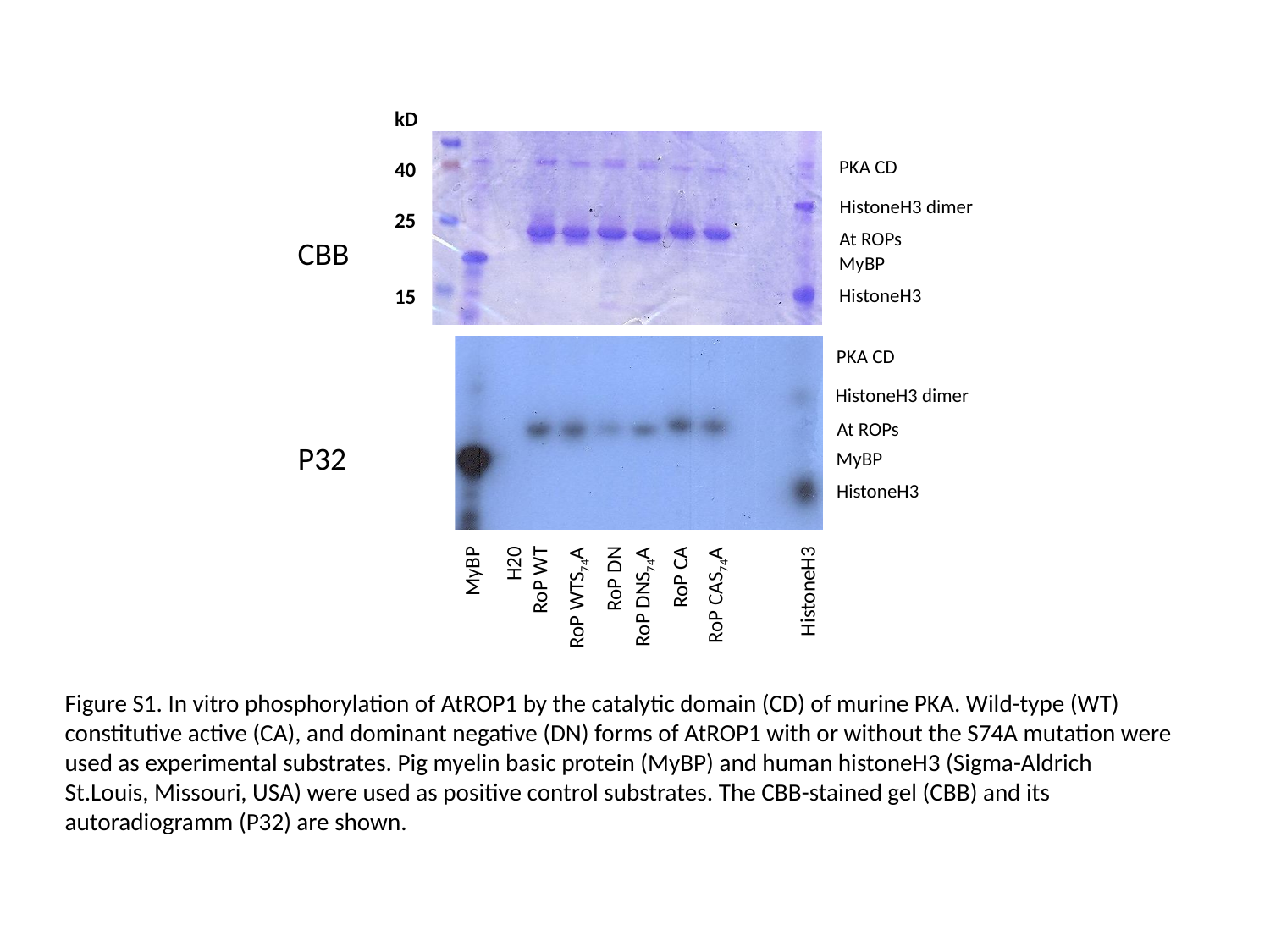

kD
40
25
15
PKA CD
HistoneH3 dimer
At ROPs
CBB
MyBP
HistoneH3
PKA CD
HistoneH3 dimer
At ROPs
P32
MyBP
HistoneH3
H20
MyBP
RoP CA
RoP DN
RoP WT
HistoneH3
RoP CAS74A
RoP DNS74A
RoP WTS74A
Figure S1. In vitro phosphorylation of AtROP1 by the catalytic domain (CD) of murine PKA. Wild-type (WT) constitutive active (CA), and dominant negative (DN) forms of AtROP1 with or without the S74A mutation were used as experimental substrates. Pig myelin basic protein (MyBP) and human histoneH3 (Sigma-Aldrich St.Louis, Missouri, USA) were used as positive control substrates. The CBB-stained gel (CBB) and its autoradiogramm (P32) are shown.
