# Supplementary material for: The Arabidopsis Rho of Plants GTPase ROP1 Is a Potential Calcium-Dependent Protein Kinase (CDPK) Substrate
Source: Plants (Basel). 2021 Sep 29;10(10):2053. doi: 10.3390/plants10102053 (PMC8539224; doi:10.3390/plants10102053)
Supplement: Supplementary file 1 [file plants-10-02053-s001.zip › Figure S2.pptx]

## Slide 1
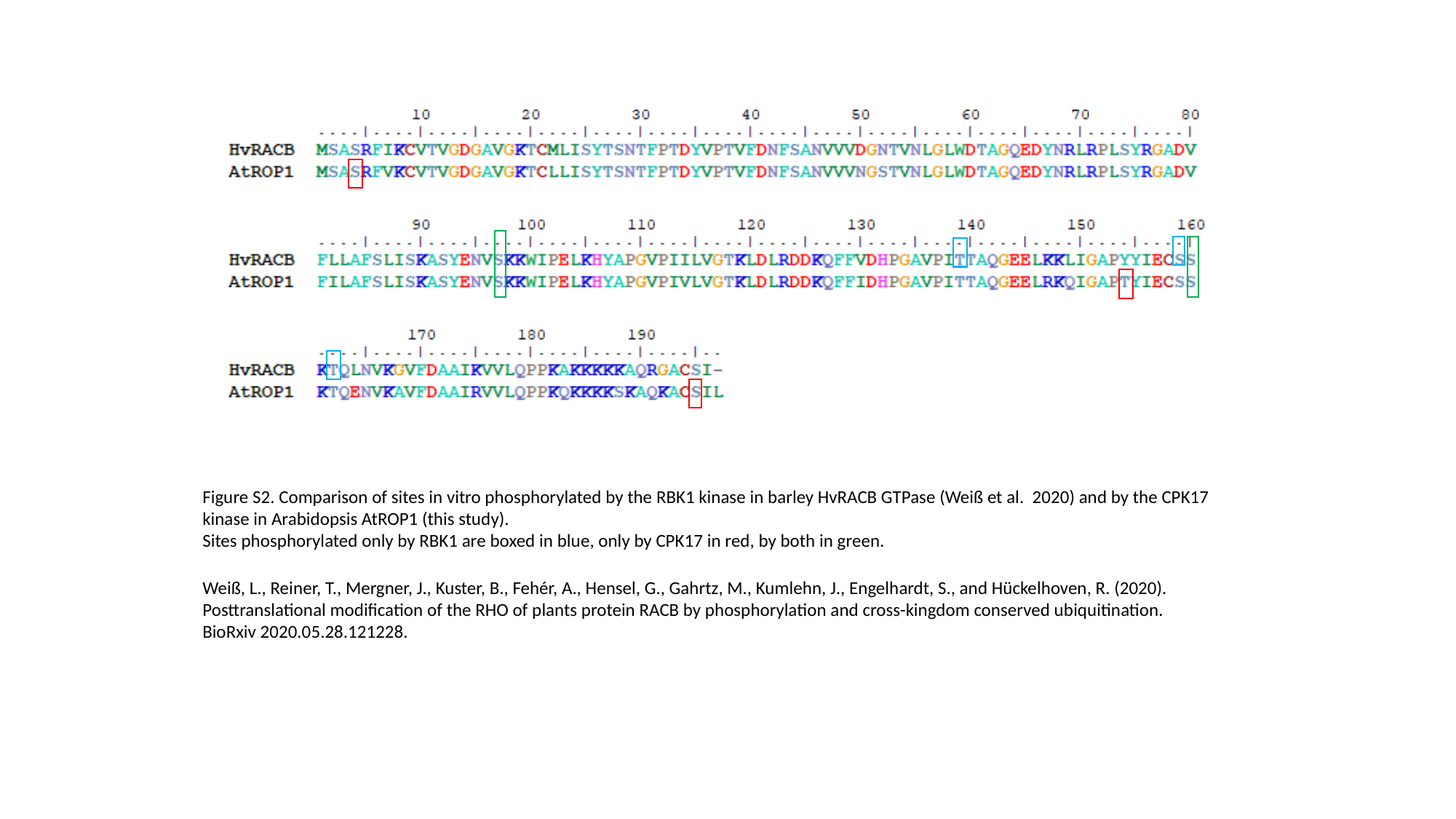

Figure S2. Comparison of sites in vitro phosphorylated by the RBK1 kinase in barley HvRACB GTPase (Weiß et al. 2020) and by the CPK17 kinase in Arabidopsis AtROP1 (this study).
Sites phosphorylated only by RBK1 are boxed in blue, only by CPK17 in red, by both in green.
Weiß, L., Reiner, T., Mergner, J., Kuster, B., Fehér, A., Hensel, G., Gahrtz, M., Kumlehn, J., Engelhardt, S., and Hückelhoven, R. (2020). Posttranslational modification of the RHO of plants protein RACB by phosphorylation and cross-kingdom conserved ubiquitination. BioRxiv 2020.05.28.121228.
