# Supplementary material for: The Arabidopsis Rho of Plants GTPase ROP1 Is a Potential Calcium-Dependent Protein Kinase (CDPK) Substrate
Source: Plants (Basel). 2021 Sep 29;10(10):2053. doi: 10.3390/plants10102053 (PMC8539224; doi:10.3390/plants10102053)
Supplement: Supplementary file 1 [file plants-10-02053-s001.zip › Table S2.pdf]

Supplementary Table S2. PCR primers used in the study

AtROP1 cloning:
>5' AtR1 NdeI
ATACATATGAGCGCTTCGAGGTTCGTAAA
>AtRop1-SalI(lm) Rev
GCAGTCGACTCATAGAATGGATCCTGCCTTCTG
>AtROP1S97A BamHI Rev
CGGGATCCACTTTTTGGCGACGTTTTCATAAC
>AtROP1S97E BamHI Rev
CGGGATCCACTTTTTCTCGACGTTTTCATAACTGG
>AtRop1 Fw (SalI + 2ncl)
GCAGTCGACATATGAGCGCTTCGAGGT
>AtROP1 Rev (Kpnl)
GCAGGTACCTCATAGAATGGAGCATGC

AtCPK17/34 cloning:
CPK17 NdeI Fw
TCCATATGGGAAATTGTTGCTCTC
CPK17 NotI Rev
TTGCGGCCGCTTTGAATGAAAGTTCACG
CPK34 NdeI Fw
TCCATATGGGAAATTGTTGCTC
CPK34 NotI Rev
TTGCGGCCGCTTTGAATGATAGTTCACGC

AtGEF2 cloning:
GEF2 EcoRI Fw
CCGAATTCATGGAGAATTTGCCAAATC
GEF2 XhoI Rev
CCCTCGAGTTCTTCTCCTCTCATTGTTTTGTC

AtRIC2 cloning:
RIC2 EcoRI Fw
GGGAATTCATGGACAGAAACGGTGC
RIC2 XhoI Rev
GGCTCGAGGACGACGGTGCCGGTGAG
